# Palladium on Plastic Substrates for Plasmonic Devices

**DOI:** 10.3390/s150101138

**Published:** 2015-01-09

**Authors:** Paola Zuppella, Elisabetta Pasqualotto, Sara Zuccon, Francesca Gerlin, Alain Jody Corso, Matteo Scaramuzza, Alessandro De Toni, Alessandro Paccagnella, Maria Guglielmina Pelizzo

**Affiliations:** 1 CNR – IFN UOS Padova, Via Trasea, Padova 7 35131, Italy; E-Mails: sara.zuccon@pd.ifn.cnr.it (S.Z.); fra.gerlin@gmail.com (F.G.); alain.corso@pd.ifn.cnr.it (A.J.C.); 2 Department of Biomedical Sciences, University of Padua, Via Ugo Bassi 58/B, Padova 35131, Italy; E-Mail: pasqualotto@dei.unipd.it; 3 ARC-Centro Ricerche Applicate, Via della Navigazione Interna 51, Padova 35129, Italy; E-Mails: matteo.scaramuzza@arc-projects.it(M.S.); alessandro.detoni@arc-project.it (A.D.T.); 4 Department of Information Engineering, University of Padova, Via Gradenigo 6/B, Padova 35131, Italy; E-Mail: alessandro.paccagnella@dei.unipd.it

**Keywords:** surface plasmon resonance, Kretschmann configuration, palladium, polycarbonate, DNA

## Abstract

Innovative chips based on palladium thin films deposited on plastic substrates have been tested in the Kretschmann surface plasmon resonance (SPR) configuration. The new chips combine the advantages of a plastic support that is interesting and commercially appealing and the physical properties of palladium, showing inverted surface plasmon resonance (ISPR). The detection of DNA chains has been selected as the target of the experiment, since it can be applied to several medical early diagnostic tools, such as different biomarkers of cancers or cystic fibrosis. The results are encouraging for the use of palladium in SPR-based sensors of interest for both the advancement of biodevices and the development of hydrogen sensors.

## 1. Introduction

Biosensors can be thought as analytical tools that convert a biological response into electrical or optical signals. It is a continually evolving sector that attempts to fulfill the demands of science and technology requiring affinity-based, label-free devices yielding real-time information on biomolecules' interactions and analyte concentrations in a large variety of applications, such as food, agricultural, nutraceutical, or environmental samples [1]. Biodevices based on surface plasmon resonance (SPR) are of great interest in this context. They show undoubted advantages, such as the extreme rapidity of analysis, the high reproducibility and sensitivity. Their application involves many scientific areas where a high performance and non-invasive method is required [2]. The physical phenomenon behind SPR tools is the excitation of surface plasmons [3]. This phenomenon occurs when TM-polarized light hits a metal film at the interface of media with different refractive indices. The interesting thing is that a slight change at the interface may lead to a change in the SPR readout, allowing real-time precise measurements of thin film properties as well as surface molecular interactions [4,5]. One of the most common optical schemes is the Kretschmann configuration, in which the light is focused onto the metal layer through a glass prism and the subsequent reflection is detected. At the resonance angle, the plasmons resonate with the incident light and a complete attenuation of the reflected beam is usually observed [1]. The SPR signal depends intimately on the materials and their contingent chemi-physical states, which are converted to detectable observables by the transducer. The role of the transducer is critical since it transforms properties and dynamics of the analyte into changes of the refractive index optically determined by investigating the position and the shape of the resonance peak [4,5]. Although the SPR technique is well established and marketed commercially, many efforts are still being focused on improving the available devices and the development of new high-performance chips is one of the main tasks in this area. Standard SPR chips consist of gold and/or silver layers on a glass support [5,6]. The choice of the substrates and the metal layers, often finely engineered, provides two paths for improvement. The use of plastic substrates such as polymethyl methacrylate, polystyrene and polycarbonate, represents a viable alternative for the reduction of the costs and for the fabrication of new mass-produced devices, compatible with polymer processing [7,8]. Plastic SPR chips based on gold and silver have been already standardized, but other metals should be investigated too. Metals such as platinum, tungsten, nickel and palladium are interesting not only for biosensing purposes, but also for gas sensing, since they all exhibit electrical and optical changes under exposure to specific gases [9,10]. In particular palladium is a specific detector for H_2_. Recent literature demonstrates the advantages in the use of such a metal for high sensitivity hydrogen sensing [11,12], also in combination with functionalization of graphene [13]. Further, palladium is interesting in its SPR response because it supports the so-called inverted surface plasmon resonance (ISPR) [14], an effect in which the thin metal exhibits a maximum at the resonance in place of a minimum.

The advantages of inverted resonance signals has been recently investigated both from a theoretical and an experimental point of view [15,16]. In the present work we demonstrate the feasibility of low cost plastic substrate chips based on palladium thin films. The chips were characterized before and after the metal deposition by using atomic force microscopy (AFM) to monitor the morphology and the roughness and to identify and optimize the fabrication procedure. The detection of a DNA single strain (ss-DNA) composed of 34 bases was selected as the target experiment and the final performances of the biosensor were tested in the Kretschmann configuration on an optical bench. The results represent a valuable proof of concept towards the development of innovative chips.

## 2. Experiments

**Figure 1.**
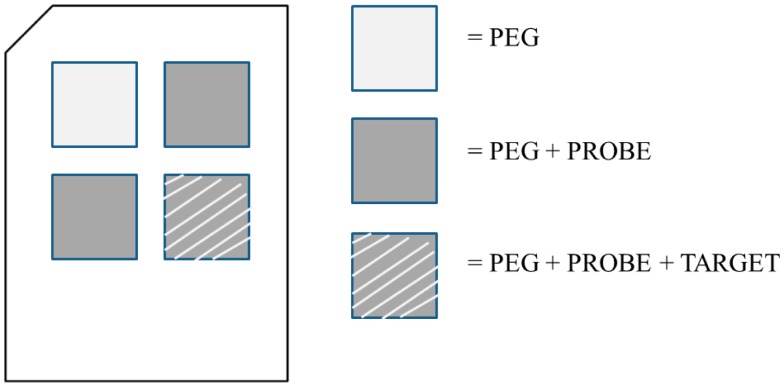
Schematic representation of the samples.

Polycarbonate substrates with a thickness of 600 μm were ultrasonically cleaned before being coated by a palladium film. The metal (palladium 99.95% pure, Kurt J. Lesker Company^®^, Clairton, PA, USA), whose thickness is optimized for SPR analysis [15,16], was deposited by electron-beam vapor deposition with a Temescal electron gun (IONVAC, Roma, Italy). The base pressure in the chamber was maintained in the range of 10^−4^ Pa during the deposition, while the chamber temperature was not increased. The growth rate of the films was controlled by a quartz crystal microbalance and the final thickness measured by using a stylus profilometer (KLA Tencor P–16+ Profiler, Milpitas, CA, USA). For our purposes, a 14 nm palladium film was deposited, as suggested from theoretical evaluations [16]. The metal adherence was verified by a scratch test. The metal-coated substrates were cleaned with ethanol and then with double distilled water and dried under N_2_ flux. No piranha solution was used in order to avoid damage to the palladium layer. Chips were functionalized with poly(ethylene glycol) (PEG) 2-mercaptoethyl ether acetic acid, Mw 3.4 kDa by a 24 h immersion in a PEG 1 mM solution. The functionalization layer was activated through EDC-sNHS 5 mM in 1× pH 6.0 MES buffer for 15 min and then the substrates were rinsed with double distilled water and dried under N_2_ flux. The activation allows one to expose the PEG carboxyl groups, able to bind with probes' amines. Through a functionalization mask the probe, *i.e.*, the ss-DNA, was immobilized over the PEG in three areas, by drop-casting of ss-DNA 20 mM solution (printing buffer 1X: 75 mM sodium phosphate, 0.005% Triton, pH 8.5), 50 μL for each cell of the functionalization mask. After 24 h, the substrates were rinsed with double distilled water and dried under N_2_ flux. Empty areas were blocked with a blocking solution (0.1 M Tris, 50 mM ethanolamine, pH 9.0) at 303.15 K for 20 min. The substrates were rinsed in washing solution (SSC 4X, SDS 0.1%) at 303.15 K, gently shaking for 20 min, then rinsed with water and dried under N_2_ flux. One area has been exposed to complementary ss-DNA in buffer SSC 4X, BSA 1 mg/mL, SDS 0.1%, formamide 50%, for 3 h at room temperature. The substrates were rinsed in SSC 1X sds 0.1% for 5 min, then in SSC 0.2X for 2 min, in SSC 0.1X for 2 min and finally in double distilled water for 30 s and dried under N_2_ flux. Figure 1 shows a map of the sample by identifying the different areas. The SPR measurements were performed by comparing adjacent wells (Figure 1).

In this experiment, the chips were carefully characterized, since there is no literature reference describing a standard procedure for the deposition of palladium onto plastic substrates. A multi-location morphological scanning of plastic and glass samples prior and after palladium deposition was performed in ambient atmosphere by using an XE-70 AFM Park System (AFM Park System, Suwon, Korea) in Non-Contact (NC-AFM) mode. In NC-AFM, the tip is held immediately above the surface and it measures the surface topography via deflections caused by longer-range attractive interactions. The absence of repulsive forces allows to softly scan the samples reducing the risk of breaking them. The images in the following section represent 5 μm × 5 μm scan areas. The roughness values, the related standard deviations and coefficients of variation are given by the statistical processing of the data obtained on multiple scans.

**Figure 2.**
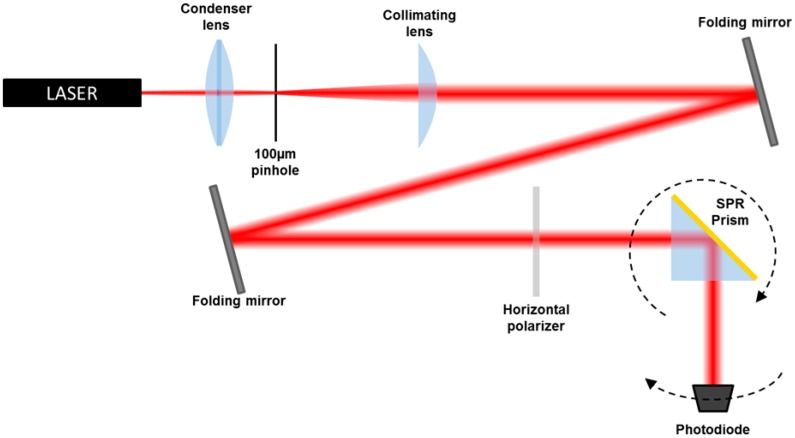
Experimental Kretschmann set-up layout.

The Kretschmann optical setup (Figure 2) for SPR measurements consists of a He-Ne laser source, λ = 632.8 nm, and a set of optical components to spatially clean the beam, followed by two folding mirrors and a polarizer [16]. The chips produced by applying the described procedure have been coupled via cargille index matching fluid to the base of a BK7 prism.

The TM-polarized laser light beam impinges the prism-sample couple and the reflected intensity is detected by a photodiode. The prism and the photodiode are mounted on a θ–2θ rotation platform, providing a Δθ resolution of 0.05° (0.9 mrad). The detector is connected to a data acquisition device (DAQ, National Instruments, Austin, TX, USA), which transfers the data to the PC. The set-up is controlled by a Labview program, which moves the rotators and stores the data in a file. In a typical SPR experiment, the reflectance of the metal layer is plotted against the incidence angle. In order to set the experiment and to analyze the data, we used the transfer matrix formalism to model the response of the device in the Kretschmann configuration. The mathematical details of these computations were already described in [2,3]. The total reflection of a TM-polarized light beam for a N-layer system is expressed by:

$$R={\left|\frac{\left(M_{11}+M_{12}q_{N}\right)q_{1}-\left(M_{21}+M_{22}q_{N}\right)}{\left(M_{11}+M_{12}q_{N}\right)q_{1}+\left(M_{21}+M_{22}q_{N}\right)}\right|}^{2}$$

where *M_i,j_* are the elements of the characteristic matrix of the chip layers structure, which is given by:

$$M_{ij}={\left(\prod_{k}^{N-1}M_{k}\right)}_{ij},i,j=1,2,$$

where:

$$M_{k}=\left[\begin{array}{cc} \cos\beta_{k} & -i\sin\beta_{k}/q_{k}\\ -iq_{k}\sin\beta_{k} & \cos\beta_{k} \end{array}\right]$$

β*_k_* and *q_k_* depend on the angle θ of incidence of light with the normal to the interface, the refractive index of the first medium (*n*_1_), the dielectric constant and the thickness of each layer (ε*_k_*, *d_k_* respectively) and the wavelength of light in vacuum (*λ*_0_). The dependence is expressed as:

$$q_{k}=\frac{{\left(\varepsilon_{k}-n_{1}^{2}\sin^{2}\theta \right)}^{1/2}}{\varepsilon_{k}}$$

$$\beta_{k}=d_{k}\left(\frac{2\pi }{\lambda_{0}}\right){\left(\varepsilon_{k}-n_{1}^{2}\sin^{2}\theta \right)}^{1/2}$$

In this experiment the first layer is BK7 glass (corresponding to the Kretschmann prism glass) (*n*_1_ = 1.515) of infinite thickness followed by a thin layer of cargille (*n*_2_ = 1.518), 600 μm polycarbonate (*n*_3_ = 1.580), palladium (*n*_3_ = 1.769, *k*_3_ = 4.292), PEG (*n*_4_ = 1.450), probe (*n*_5_ = 1.450), target (*n*_6_ = 1.450) and finally the medium (air, *n*_7_ = 1) of infinite thickness. The theoretical analysis, the experimental results concerning the new chip development and the SPR response are described in the following section.

## 3. Results and Discussion

Plastic prototype SPR chips based on palladium coating onto polycarbonate supports were fabricated and a proof of concept experiment realized. The fabrication process and validation of the method were verified step by step to prove the feasibility of the new chips. While the deposition and the adhesion onto plastic substrates of standard metals are well established know-how [17], the procedure has not yet been standardized for palladium and the described results are a first such attempt. If we consider gold, for example, the adhesion issues are solved by inserting a chromium layer between the substrate and the metallic film [18]; such a bi-layer has been proven to withstand piranha treatment and offers long term stability. On the contrary, from our experience, palladium directly deposited onto plastic cannot stand aggressive cleaning cycles like piranha treatments, suggesting the need of an adhesion-promoting layer.

**Figure 3.**
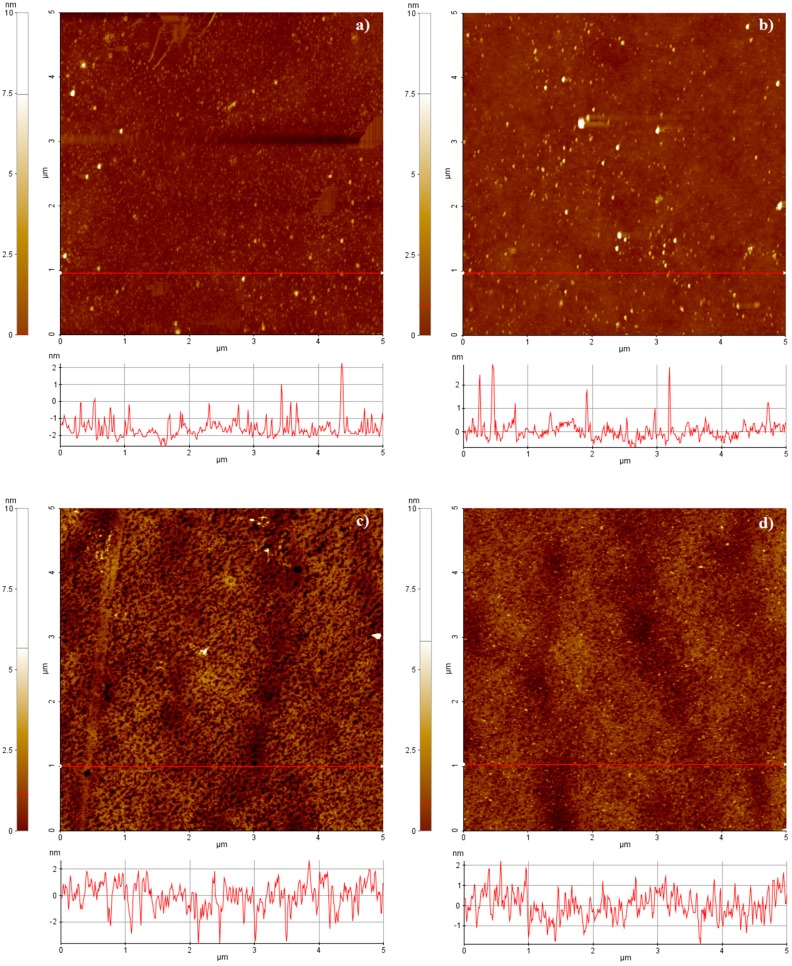
AFM morphologies: (**a**) glass; (**b**) Pd_glass; (**c**) polycarbonate; (**d**) Pd_polycarbonate. The selected images are representative of the samples set.

**Figure 4.**
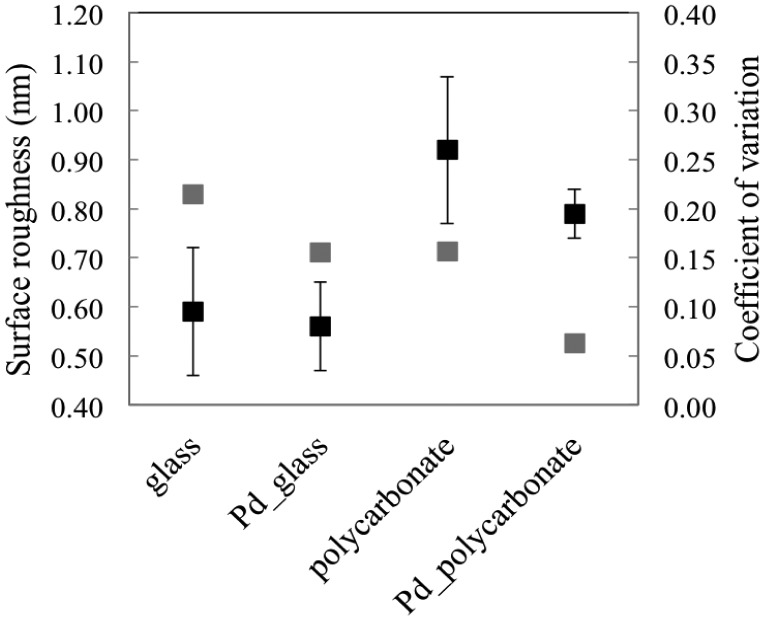
Black markers: mean roughness with error bars of the pristine samples and palladium coated chips (primary vertical axis); gray markers: coefficient of variation of the mean roughness (secondary vertical axis).

In this specific work, the functionalization of the samples was performed without piranha procedures, thus reducing the time laps between the deposition and the biological functionalization, with care to protect the metal from potential sources of contamination during handling and storage. Many parameters of the coatings were monitored. The surface roughness of the samples was deeply investigated; the specimens were characterized from a morphological point of view before and after the metal depositions. The pristine and metalized topographies are shown in Figure 3. The local average roughness of the palladium film onto plastic support is 0.79 nm, 15% lower than the pristine substrate (Figure 4). The coefficient of variation of such measurement is lower than the bare plastic by confirming the palladium property to smooth the surface of the nude support (Figure 4, gray markers). This trend, although less pronounced as demonstrated by the statistical parameters, was also observed for standard samples of palladium onto glass (Figure 4). Unlike other metals, including gold [18], palladium seems to smooth the virgin sample surface, thus improving the quality of the pristine specimen before functionalization.

Once the characterization was performed, the chip was mounted in an SPR platform to perform an experimental feasibility test. Proper simulations were performed to predict the response of the set-up. The DNA single strain was modeled as a dielectric layer of only few nanometers thickness and refractive index *n* = 1.450 [19–21]. The theoretical angular shift predicted by the model is Δθ = 0.15°−0.20° (2.5−3.5 mrad) depending on the adopted input parameters target description [19–21].

**Figure 5.**
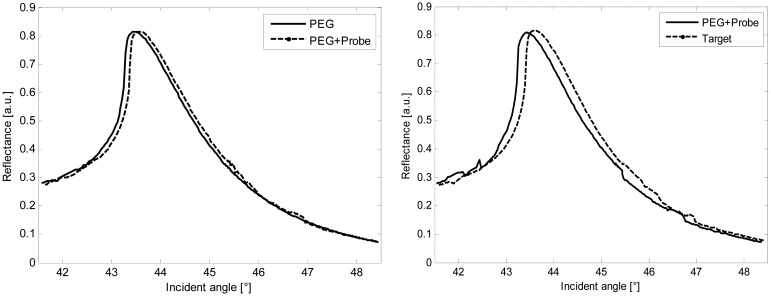
SPR responses; each measurement refers to two adjacent wells (Figure 1). **Left**: the plasmon resonance of the palladium + PEG has been compared to the response of palladium +PEG + probe; **Right**: the plasmon resonance of the palladium + PEG + probe has been compared to the response of the target.

Figure 5 shows the SPR detection of the ss-DNA performed by using the palladium plastic chip in the SPR set-up. The curves refer to the functionalization, the immobilization of the probe and the target, respectively. Specifically, the experimental angular spread between the ss-DNA probe and its complementary is 0.20° (3.5 mrad), then the presence of the target is perfectly detected by the SPR device in accordance with the theoretical values (Table 1).

Although a standard gold-based chip shows in general higher sensitivity $\left(\Delta S_{th}=\frac{s_{Pd}}{s_{Au}}=0.8\right)$, the advantage of using palladium is essentially due to the peculiar maximum resonance. The peak is very sharp, and thus easier to detect in any experimental conditions independently of the noise background [15,16]. These properties are analytically summarized by the quality factor $Q=\frac{c}{\text{FWHM}}$ of the curve, thus defined as the ratio between the contrast *C* and the full width at half maximum *FWHM* [15]. Higher *Q* values are desirable to improve the measurement accuracy. An experimental value of 0.37 (deg)^−1^ (21.26 rad^−1^) was found for all the palladium SPR signals, confirming that the curve profile of such a metal does not worsen even when the detection of the target is accomplished. The theoretical *Q* factor was also estimated for a classic gold transducer, that shows lower values than in palladium SPR response (Q_PEG_ = 0.186 deg^−1^ (10.70 rad^−1^) for PEG detection, for Q_target_ = 0.156 deg^−1^ (8.96 rad^−1^) target detection). The comparison confirms results already discussed [15,16], in which gold and palladium have been theoretically analyzed in terms of sensitivity, quality factor and general performances [15,16]. In some cases the characteristics of the palladium make it preferable to gold [15,16] by improving the measurement accuracy. In conclusion, all the results show that the new chips based on plastic and palladium can be successfully adopted as SPR transducers.

## 4. Conclusions

Exploration of new metals for specific SPR applications moves towards a device performance optimization. The use of palladium is attractive, as it offers new perspectives, not only for biological applications, but also for development of hydrogen sensing applications and industrial purposes. The combination of palladium and plastic substrates may be of great interest for large consumer and industrial applications. The results proof the viability of such a path, validating both the chip fabrication procedure as well as the performance of a proof of concept prototype system.

## Figures and Tables

**Table 1.** SPR measurements: angular shifts of adjacent wells.

**Target**	**Experimental Angular Shift**	**Theoretical Angular Shift**
PEG—PEG + Probe	0.15° ± 0.05° (2.5 ± 0.9 mrad)	0.15°–0.20° (2.5–3.5 mrad)
PEG + Probe—PEG + Probe + Target	0.20° ± 0.05° (3.5 ± 0.9 mrad)	0.10°–0.15° (1.8–2.5 mrad)
